# Uncommon presentation of a common disorder: Syncope with AVNRT in setting of a structural anomaly

**DOI:** 10.1002/ccr3.2674

**Published:** 2020-01-31

**Authors:** Paul Jae‐Hyuk Lee, Paul Varosy, Amneet Sandhu

**Affiliations:** ^1^ Department of Internal Medicine University of Colorado Aurora Colorado; ^2^ Section of Electrophysiology Division of Cardiology University of Colorado Aurora Colorado; ^3^ Section of Cardiology Eastern Colorado VA Medical Center Aurora Colorado

**Keywords:** AV nodal re‐entrant tachycardia, cryoablation, electrophysiology, intracardiac echocardiography, syncope, tachyarrhythmia

## Abstract

Syncope in setting of AV nodal re‐entrant tachycardia should prompt the clinician to perform further workup for comorbid conditions, including structural cardiac abnormalities. In complex cases, ICE may be helpful in delineating the anatomy to facilitate safe ablations.

## Graphical abstract

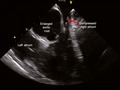

## 1 INTRODUCTION

AV nodal re‐entrant tachycardia (AVNRT) is the most common clinically relevant supraventricular tachycardia (SVT). Dual AV nodal physiology, substrate required for AVNRT, is present in approximately 20%‐30% of the population though only a minority of such patients suffers from symptomatic tachycardia.^1^ Importantly, syncope is rarely attributable to AVNRT outside of concurrent structural heart disease or comorbid conditions. In this report, we present a case of a patient who presented with syncopal episodes found to be associated with episodes of AVNRT, secondary to his tachyarrhythmia in the context of distorted cardiac anatomy discovered with use of intracardiac echocardiography (ICE).

## 2 CLINICAL CASE

**Figure 1:**
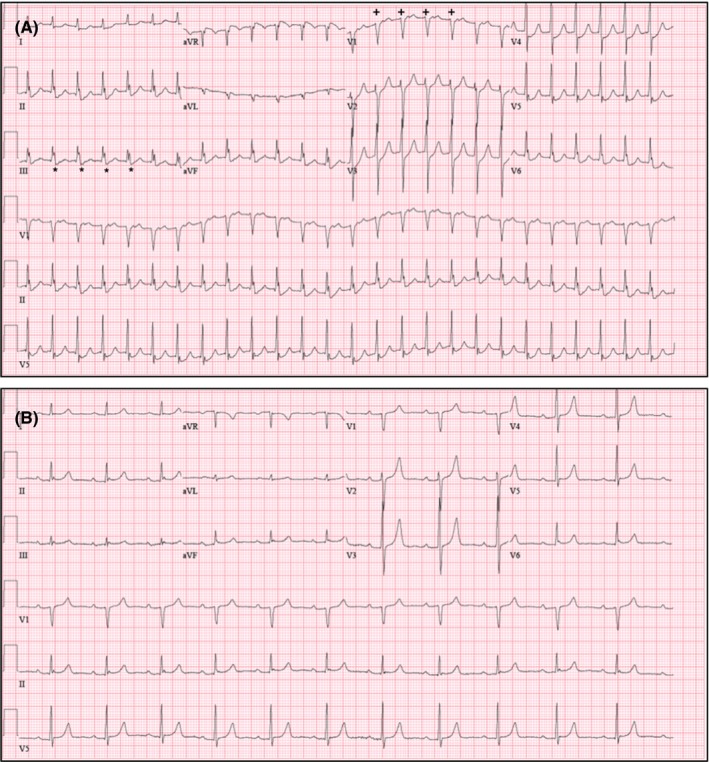
A, Preablation with rate of approximately 160 BPM with pseudo‐S(*) and pseudo‐R′ waves(+) on leads III and V1, respectively. B, Postablation with return to normal sinus rhythm with baseline prolonged P‐R interval

**Figure 2:**
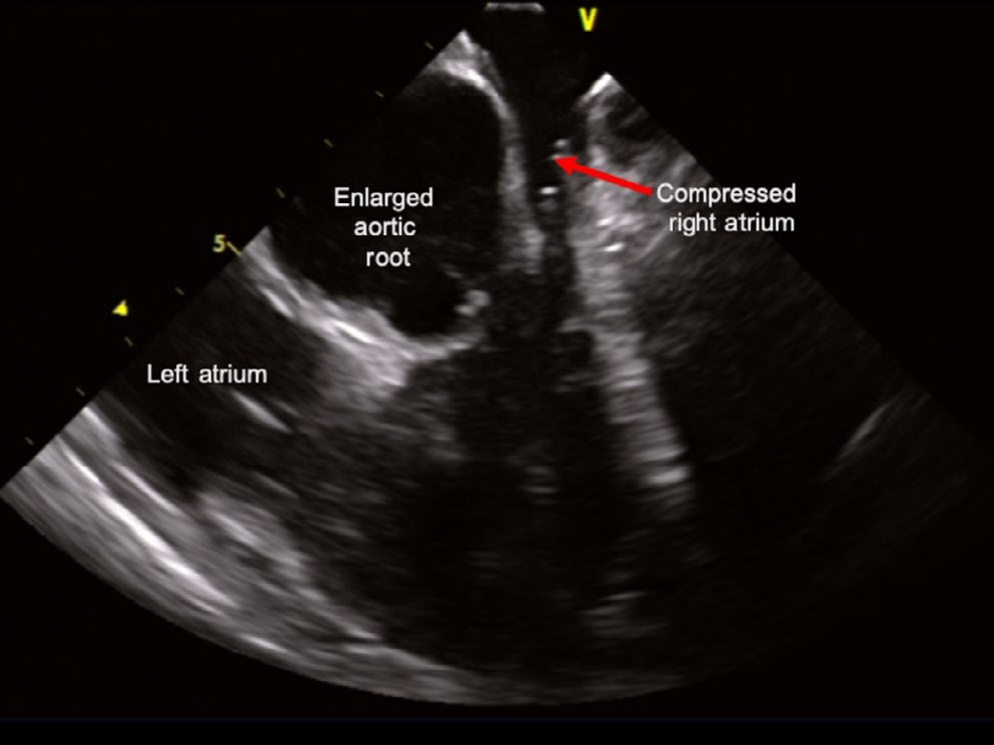
ICE image captured during cryotherapy revealing prominent aortic root, with diminished RA size

**Figure 3:**
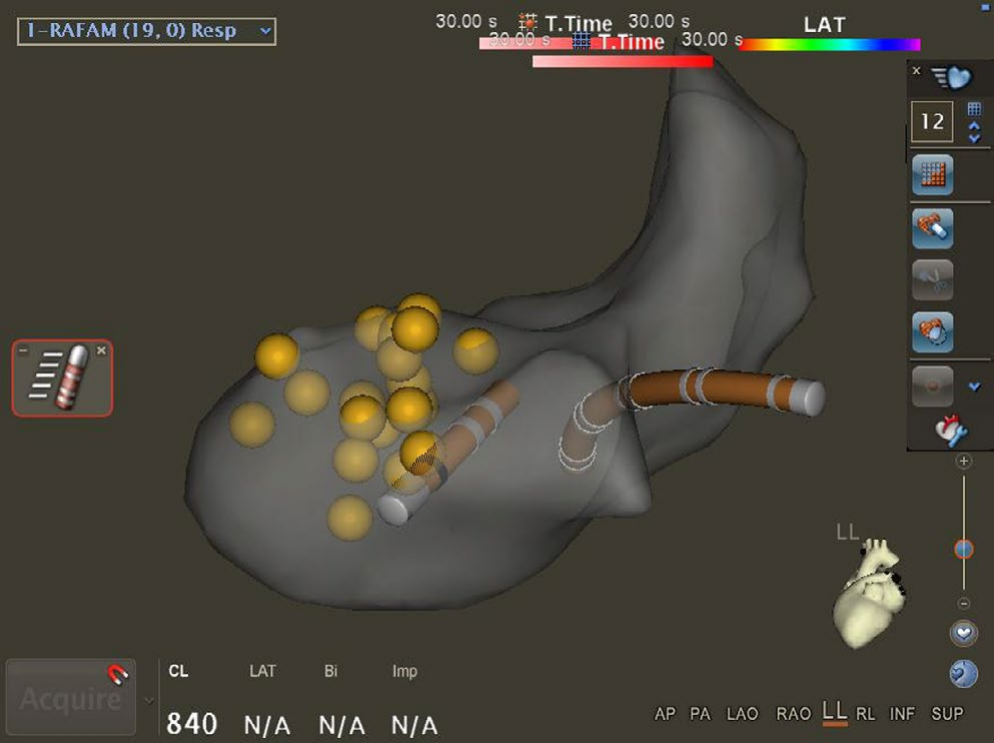
Left lateral view of the RA during cryoablation therapy. Note placement of the cryoablation catheter (Left with black stripe) near the His/native conduction system (Yellow) in relation to the decapolar catheter in the coronary sinus (Right)

A 69‐year‐old man without prior cardiovascular events, presented with new‐onset SVT resulting in shortness of breath, dizziness, and multiple syncopal episodes over the course of 1‐year. Cardiovascular medicines included amlodipine, atenolol, and doxazosin for treatment of hypertension though adjustment of these medicines did not mitigate syncopal episodes. Other etiologies, including autonomic dysfunction, reflex syncope, orthostatic hypotension, were also considered but given the patient's history and description of the patient's symptomology, it deemed highly unlikely. Physical examination was grossly benign at presentation—nontoxic appearing with cardiovascular examination revealing regular rate and rhythm without any extra heart sounds. Workup with ECG was concerning for SVT (Figure 1A), which was successfully terminated with administration of IV adenosine. Transthoracic echocardiogram (TTE) showed normal ejection fraction without evidence of wall motion or structural abnormalities. A nuclear stress test was undertaken without suggestion of myocardial ischemia or infarction. Given repeated recurrences of tachycardia and syncope, the patient was hospitalized for expedited workup. Electrophysiologic (EP) study revealed a diagnosis of “typical,” slow‐fast AVNRT. Attempt at slow pathway modification as an invasive treatment for typical AVNRT revealed significant anatomic distortion with a diffuse His cloud, a large and funneling coronary sinus ostium, prominent eustachian ridge, and aortic knob. Due to the close proximity of slow pathway fibers to native conduction system, limited RF ablation was conducted, resulting in failed attempts to treat the patient's tachyarrhythmia. Subsequently, the patient was arranged for repeat EP study with use of ICE and consideration of cryoablation to minimize the risk of injury to his native conduction system. On follow‐up EP study, ICE was used to visualize the region of interest. This revealed a prominent aortic root resulting in significant distortion of the right atrium (RA; Figure 2). Given minimal RA volume, this finding explained the patient's syncope and presyncope secondary to sustained tachyarrhythmia. Under ICE guidance, the cryocatheter was introduced and positioned in the region of interest. While monitoring native conduction and under surveillance of catheter position under ICE, successful cryocatheter‐guided ablation was conducted along the slow pathway rendering the SVT noninducible (Figure 3). The technique of cryomapping was employed—the catheter was cooled to −30°C to allow for testing—at this level, we confirmed that slow pathway conduction was suppressed, but fast pathway conduction remained intact before proceeding to a full 4‐minute cryoablation at −70°C. The patient remains arrhythmia‐free on follow‐up without episodes of syncope having regained quality of life.

## 3 DISCUSSION

Although there is a myriad of different causes of syncope,^2^ it is widely accepted that true syncope primarily due to SVT is a rare phenomenon.^3^ Common symptoms associated with SVT include dizziness, shortness of breath, symptomatic palpitations with chest pain, and/or anxiety. It has been previously suggested that that tachycardia greater than 170 beats per minute may increase the risk for syncope,^3^ but this has been disputed given the lack of association between reported syncope and tachycardia cycle length.^4^, ^5^, ^6^, ^7^ Therefore, it is prudent that clinicians be cognizant of other factors that may contribute to syncope in a patient presenting with AVNRT.

Accordingly, multiple case studies reporting syncope in setting of AVNRT with other comorbid conditions have been published.^8^, ^9^, ^10^ Compared to younger cohorts, patients with advanced age were also found to have increased frequency of syncope.^6^ This may be explained by frailty, but also by the higher prevalence of structural cardiac disease in the elderly. Independently, AVNRT may result in dysregulated vasomotor response,^7^ and AV dissociation, interfering with the normal systolic and diastolic function and ultimately decrease cardiac output—predisposing to syncope. Collectively, these findings indicate that syncope may preferentially occur in the setting of another concomitant disorder or structural heart disease, which negatively affects cardiac output.

In our patient, we hypothesize that the syncopal episodes were a result of RA compression due to an enlarged aortic root, resulting in underfilling of the right ventricle during episodes of AVNRT (Figure 2). Another possibility includes an anomaly in the conduction system caused by the structural changes elicited by the RA distortion. Importantly, evaluation of cardiac structures and function with TTE prior to the EP study was significant only for prominent thickening at the septal base without outflow obstruction. Routine, surface TTE did not reveal the severity of RA distortion or enlargement of the aortic root or ascending aorta as effectively ICE ultimately demonstrated, suggesting that, likely a reflection of the limitations of TTE in at least some situations.

Given multiple prior noted anatomic challenges at the time of the initial EP study, we elected to use ICE during the follow‐up procedure. Adjunctive use of ICE with fluoroscopy and electroanatomic mapping allowed direct visualization of the surrounding structures, improving catheter guidance to the ablation target within the distorted triangle of Koch. The use of ICE revealed challenges noted in the initial ablation primarily centered around RA distortion from the prominent aortic root. In addition, ICE offers other advantages over traditional methods of fluoroscopy and electroanatomic mapping systems in that it allows for real‐time anatomic evaluation, assessment of the catheter contact with the endocardium and real‐time catheter location in relation to neighboring cardiac structures. Use of ICE also minimizes the risk of VA block, as demonstrated by the results from the study by Fisher and colleagues.^11^

Our decision to use cryoablation to treat our patient was supported by proximity of slow pathway fibers to the His bundle and the baseline first‐degree AV block, both of which increase potential for damage to the native conduction system. In summary, presentation of SVT in context of syncope or presyncope should alert clinicians to consider additional workup. Complementary to TTE, ICE can serve as a valuable tool to assist with evaluation of cardiac anatomy, in particular anatomy important in relationship to electrophysiological procedures, real‐time catheter positioning, and monitoring catheter‐tissue contact. Lastly, clinicians should consider the use of ICE to assist with challenging catheter ablation procedures attempting to treat AVNRT.

## 4 CONCLUSION

We present an uncommon case of recurrent syncope and presyncope secondary to AVNRT in the setting of structural cardiac abnormality that further compromised the cardiac output. Syncope should alert clinicians to perform further workup assessing for comorbid conditions or structural heart disease. In addition to facilitating safer ablation, ICE can be useful in delineating cardiac anomalies, at times not evident by routine surface echocardiography.

## CONFLICT OF INTEREST

None declared.

## AUTHOR CONTRIBUTIONS

Paul Lee: involved in writing the article and interpretation of data. Paul Varosy: involved in critical revision of the article. Amneet Sandhu: involved in conception and design, interpretation of data, critical revision of the article, and overall responsibility of article.
